# Mitigation of eutrophication caused by wastewater discharge: A simulation-based approach

**DOI:** 10.1007/s13280-020-01346-4

**Published:** 2020-05-25

**Authors:** Michał Preisner, Elena Neverova-Dziopak, Zbigniew Kowalewski

**Affiliations:** 1 Mineral and Energy Economy Research Institute, Polish Academy of Sciences, ul. Wybickiego 7A, 31-261 Kraków, Poland; 2 AGH University of Science and Technology, al. Mickiewicza 30, 30-059 Kraków, Poland

**Keywords:** ASM simulation, Eutrophication potential, Nutrients bioavailability, Wastewater treatment

## Abstract

Mitigation of eutrophication, intensified by excessive nutrient load discharge in wastewaters regulated by restrictive legal requirements, remains one of today’s most important global problems. Despite implementation of the Water Framework Directive, the Urban Wastewater Directive and the HELCOM recommendations, the actual condition of surface water is still not satisfactory. In response to the above, the study presents an alternative approach for surface water protection against eutrophication based on the selection of appropriate nutrient removal technologies. An activated sludge model simulation was used to enable the identification of environmentally justified nutrient removal systems with lowest eutrophication potential of treated wastewater conditioned by bioavailable nutrient forms content. Based on the outcome of the study, the 3-stage Bardenpho system was identified as the most efficient for bioavailable phosphorus removal, while the Johannesburg system proved to have the highest efficiency for bioavailable nitrogen removal. The proposed eutrophication mitigation approach underlines the need for a reconsideration of current legal regulations which ignore nutrient bioavailability and key eutrophication limiting factors.

## Introduction

The problem of anthropogenic eutrophication of surface waters was first identified in the 1970–1980s of the twentieth century (Bartosova et al. 2019) and continues to be a global problem leading to an imbalance of all water types and a significant deterioration of the ecosystem services provided by aquatic systems (Schindler 2006; Brauman et al. 2007; Grizzetti et al. 2015). The eutrophication intensification is very closely associated with an increase of nutrient anthropogenic loads consisting mainly of nitrogen and phosphorus which are discharged into surface waters from various sources.

Global nutrient transport in the twentieth century was dominated by agricultural sources, and from 1900 to 2000, nitrogen flow from this source increased from 19 to 51% of total nitrogen (TN) load and phosphorus flow from 35 to 56% of total phosphorus (TP) load. However, the highest increase in nitrogen and phosphorus global supply over this period was caused by point sources (mostly wastewater discharge) which increased from 2.0 to 8.0 TgN year^−1^ (340% increase) and from 0.2 to 1.0 TgP year^−1^ (500% increase), respectively (Beusen et al. 2016). The global forecast for future nutrient discharges indicates their increase from 6.4 TgN year^−1^ and 1.3 TgP year^−1^ in 2000 to 12.0–15.5 TgN year^−1^ and 2.4–3.1 TgP year^−1^ in 2050 (Van Drecht et al. 2009).

Municipal wastewaters containing large amounts of nitrogen and phosphorus compounds play a key role in water trophic state deterioration (Li et al. 2012; Smol et al. 2020b). Moreover, along with economic growth, population increase, expansion of urbanization and the resultant development of water supply and wastewater disposal systems, nutrient loads of urban origin are constantly increasing (Zhang et al. 2017; Lin et al. 2020; Smol et al. 2020a).

Consequently, the key technique for preventing eutrophication development and mitigating its negative consequences has become the reduction of the biogenic load introduced into wastewater receivers. The basic tools for achieving this goal are legal requirements introduced in all European countries establishing the conditions for wastewater discharge. These requirements are systematically becoming stricter in terms of nutrient content which has resulted in the use of advanced nutrient removal technologies in expensive and complex wastewater treatment systems.

The permissible values of basic wastewater quality parameters (TN and TP), determining allowable loads discharged into receivers, are often not adequate for understanding the impact of treated wastewater on eutrophication development in wastewater receivers. There are two reasons for this. Firstly, algae development is determined primarily by the main limiting factor, which in different surface water types can be conditioned by either phosphorus or nitrogen (Nyenje et al. 2010; Le Moal et al. 2019; Wang et al. 2019). Secondly, algae development is determined by bioavailable forms of nitrogen and phosphorus (predominantly their inorganic forms) (Conley et al. 2009; Majewski 2014; Gubelit et al. 2016; Poikane et al. 2019a) where the content of these bioavailable forms in treated wastewater determines their eutrophication potential. Based on this, the authors define the concept of “eutrophication potential of treated wastewater” (EPTW). EPTW is the degree of impact on the development of eutrophication processes conditioned by the content of bioavailable nutrient forms of wastewater discharged into the receiver (Neverova-Dziopak et al. 2019).

This knowledge about the limiting factors of eutrophication in receiving waters and the bioavailability of various nutrient forms in discharged treated wastewater should be taken into account when developing wastewater quality standards and it should determine the conditions of their discharge. However, the current approach to eutrophication control caused by wastewater discharges does not cover the above mentioned aspects and, thus, needs to be reconsidered (Pihlajamäki and Tynkkynen 2011) as it will help to understand the reasons for the failure to achieve the objectives of the Water Framework Directive (2000/60/EC) directed towards achieving good surface water status in all European Union (EU) countries by 2015 (which turned out to be a largely utopian target (EC 2019)). Without establishing reliable nutrient criteria, achieving good ecological status in all rivers, lakes, coastal and transitional waters by 2027 seems to be impossible (Poikane et al. 2019b).

In this context, this study aims to develop an alternative approach for surface water protection against eutrophication based on the selection of appropriate nutrient removal technologies.

## Approach proposed to minimize wastewater impact on eutrophication

The proposed approach is based on the following assumption: mitigation of the eutrophication process requires an ecosystem approach (Wang and Wang 2009; Wu et al. 2012) based on knowledge about inorganic nutrition mechanisms of aquatic vegetation (i.e., consideration of the bioavailability of various chemical forms of nitrogen and phosphorus) and the identification of key eutrophication limiting factors in wastewater receivers. This knowledge should be used in setting legal regulations regarding quality standards for wastewater discharge into surface waters.

Unfortunately, current legal quality standards for treated wastewater in force in the EU Member States are mainly based on TN and TP content (EC 1991; Neverova-Dziopak and Preisner 2015; Vojtěchovská Šrámková et al. 2018) with permissible nutrient concentrations set depending on the wastewater treatment plant (WWTP) capacity as expressed by population equivalent (p.e.) and the degree of the receiver’s sensitivity to eutrophication. Stricter requirements for nitrogen and phosphorus content in treated wastewater require the application of expensive and energy-intensive enhanced biological nutrient removal (EBNR) technologies (Hanmin et al. 2009; Valverde-Pérez et al. 2015; Estrada-Arriaga et al. 2016). Those technologies while meeting the criteria for efficient TN and TP removal, at the same time, generate effluents with high eutrophication potential due to the increased content of inorganic (bioavailable) forms of nutrients (Kowalewski et al. 2016).

The application of an integrated dual-nutrient reduction strategy does not always ensure a healthy balance between nitrogen and phosphorus content (Howarth and Marino 2006; Desmit et al. 2018) and it also causes an increase in operating and investment costs in the wastewater sector (Schoumans et al. 2015). This confirms the validity of considering EPTW in wastewater discharge permits because, otherwise, not even advanced technologies will be able to stop eutrophication and the process could in fact be accelerated (Cui et al. 2013; Wang et al. 2017; Zhang et al. 2019).

The weakness of currently applicable regulations is clearly visible in Poland. In the period 2000–2015, over 15 billion Euro was spent on implementing the National Municipal Wastewater Treatment Program (NMWWTP) under which 405 new WWTPs were built and 1575 were modernized (NWMH Polish Waters 2018). Unfortunately, according to the results of an assessment of surface water status in the same period, over 90% of rivers, 75% of lakes and 100% of coastal and transitional waters in Poland are still threatened by the risk of eutrophication (NWMH Polish Waters 2017) currently creating a paradoxical situation: wastewater quality standards are becoming more and more restrictive, financial expenditures for wastewater treatment and investments in this area are increasing, but the state of surface waters is still not satisfactory.

**Fig. 1:**
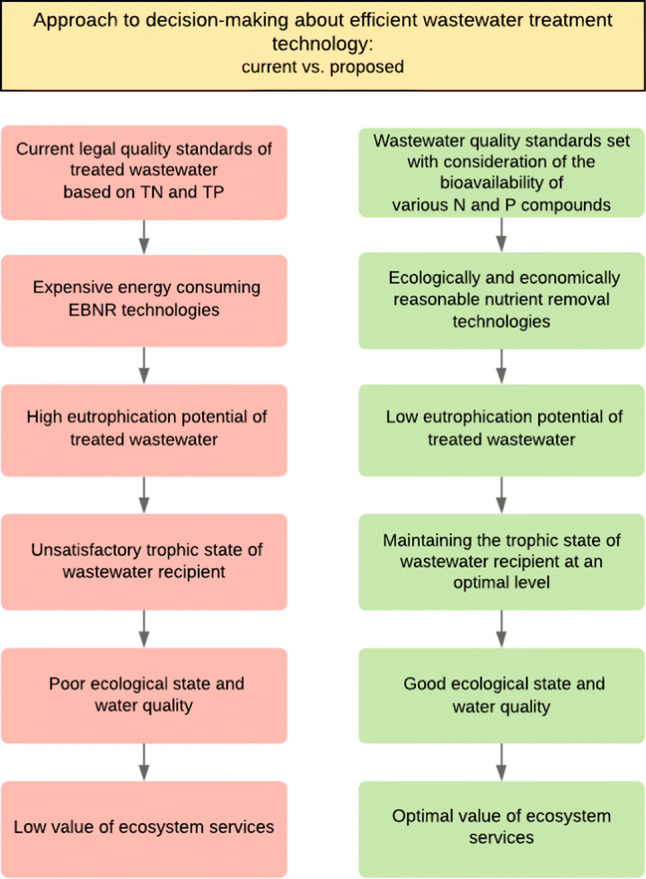
Current approach vs. proposed approach to decision-making about efficient wastewater treatment technology

The above considerations indicate the necessity for a re-evaluation of the existing approach and the development of alternatives based on a strong emphasis on ecological aspects. In this context, the authors prepared a comparison analysis of the existing approach to eutrophication management and the one they propose. The results are presented in Fig. 1.

An analysis of the current situation allows one to state that without consideration and harmonization of the key factors related to eutrophication process management and control, it will be difficult to expect tangible results in terms of improving the condition of surface waters and water consumption properties.

## Materials and methods

The research methodology included a summary of current knowledge of eutrophication process mitigation proceeded by a comprehensive literature analysis focused on eutrophication limiting factors and the efficiency of EBNR technologies. Special attention was given to the bioavailability of nutrient compounds discharged together with treated municipal wastewater. Based on an understanding of the above and using Activated Sludge Model (ASM) simulation (Makinia 2010), the authors propose an ecologically justified approach for eutrophication mitigation with a special focus on the selection of wastewater technology and the factors limiting eutrophication in the receiving ecosystem. The simulation was aimed at identifying wastewater treatment technologies with the lowest EPTW, which is determined by the share of bioavailable (inorganic) nitrogen and phosphorus compounds in treated wastewater discharged into receiving waters (Li and Brett 2015; Fan et al. 2018).

The share of bioavailable nitrogen (BAN%) and bioavailable phosphorus (BAP%) in total content was calculated according to Formulas 1 and 2 (Nakajima et al. 2006; Li and Brett 2012):

1 $${\text{BAN}}\% ={\frac{{\text{TIN}}}{{\text{TN}}}} \cdot 100\%$$

2 $${\text{BAP\% }} = \frac{{{\text{P}}_{\text{i}} }}{\text{TP}} \cdot 100\%,$$

where BAN% is the share of BAN in TN content (%), BAP% is the share of BAP in TP content (%), TIN is the inorganic N as the sum of N-NH_4_; N–NO_2_; N–NO_3_ (mg L^−1^), and P_i_ is the inorganic P as P–PO_4_ (mg L^−1^).

### Simulation assumptions and databank

To support the decision-making process regarding efficient nutrient removal technologies, an ASM-based simulation was carried out using the BioWin software developed by EnviroSim Associates Ltd., Canada. BioWin is often used by wastewater professionals to model and simulate various wastewater treatment processes (Liwarska-Bizukojc and Biernacki 2010) as it includes among other features, biological removal of organic compounds, nitrification, denitrification, chemical and biological phosphorus removal, as well as SBR reactors, membrane reactors, oxidation ditches, aerobic and anaerobic digestion of sludge, separation processes and many others (Nelson and Sidhu 2009). In our research, BioWin software version 5.1 was used for biological process modeling as it contains three ASM group activated sludge models—(ASM1, ASM2d and ASM3). In addition, it also contains models for the nitrification and transformation of sewage sludge (Vitanza et al. 2016; Elawwad et al. 2019).

Modeling of biological wastewater treatment processes can be used to select the optimal technology for nutrient removal in terms of minimal wastewater impact on eutrophication development. For the purposes of this research, the simulation was carried out using a dataset provided by the authorities of the municipal WWTP “Kujawy” in Krakow, Poland, which serves the Nowa Huta district and the surrounding area (approx. 250 000 inhabitants). It is a mechanical–biological WWTP with EBNR, using activated sludge technology optionally supported by chemical precipitation of phosphorus compounds. The receiver of treated wastewater is the Vistula River. According to the wastewater discharge permit, the effluent flow cannot exceed 100 000 m^3^ day^−1^ as an average, and 80 000 m^3^ day^−1^ during periods without rain. In the rainy season and during the snow melting period, wastewater discharge up to an amount of 9320 m^3^ h^−1^ is permitted.

**Fig. 2:**
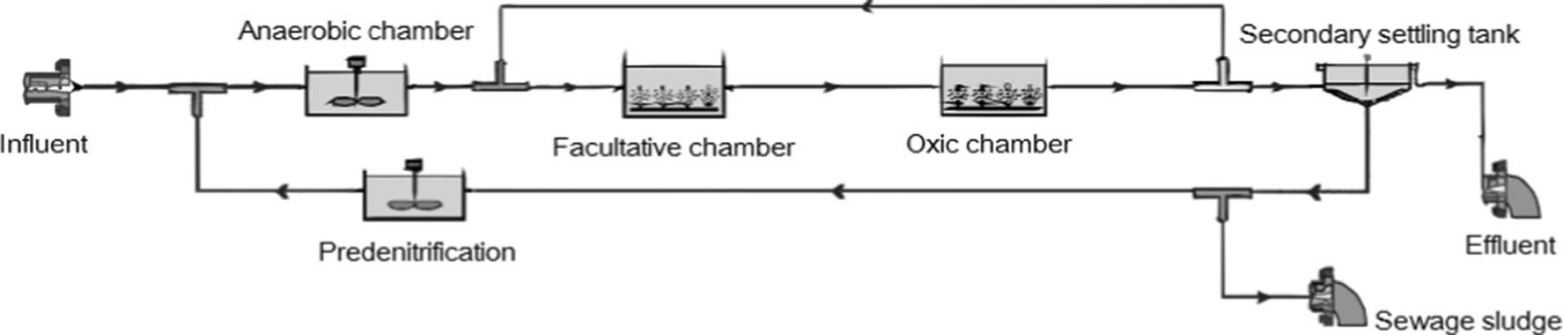
Schema of the biological treatment stage at Krakow-Kujawy WWTP (baseline setup)

The biological part of the WWTP consists of four parallel three-phase reactor systems with separated anaerobic, anoxic and oxic sections with internal recirculation, each with a capacity of 17 500 m^3^ day^−1^. Each reactor consists of 9 chambers with a depth of 4.7 m and a total capacity of 16 010 m^3^. The predenitrification chamber with a depth of 3.36 m and a capacity of 620 m^3^ is integrated within the external recirculation line. The anaerobic and facultative chambers are equipped with low-speed mixers, while the oxic chambers are additionally equipped with aerators. Currently, four technological lines of bioreactors are in operation, each equipped with two radial secondary settling tanks with scrapers having a diameter of 42 m and an active depth of 3 m (Krakow Waterworks 2013). The technological line of the biological part of the Kujawy WWTP is presented in Fig. 2.

The simulation was based on wastewater quality monitoring data from 2013, flowing into a single bioreactor system in a fixed quantity of 17 500 m^3^ day^−1^. Chemical analysis of raw and treated wastewater was carried by an accredited laboratory within the Krakow-Kujawy WWTP. The assumed simulation period of 21 days corresponded to the annual number of indicator measurements and this simplified input set is shown in Table 1.

**Table 1:** Set of the input wastewater parameters in the Krakow-Kujawy WWTP (Krakow Waterworks 2013)

Krakow-Kujawy WWTP	N–NH_4_ (mg L^−1^)	N–NO_3_ (mg L^−1^)	N–NO_2_ (mg L^−1^)	N_Kj_ (mg L^−1^)	ON (mg L^−1^)	P–PO_4_ (mg L^−1^)	TN (mg L^−1^)	TP (mg L^−1^)
Influent	47.26	0.13	0.02	67.54	20.28	3.71	67.65	6.60
Effluent	10.80	5.29	0.77	11.84	1.04	0.20	17.52	0.37

The study involved the analysis of various nutrient form contents in treated wastewater obtained in 10 activated sludge technology systems. These were conventional activated sludge (CAS), anoxic–oxic (AO), 3-stage Bardenpho, 5-stage Bardenpho, Johannesburg (JHB), modified Johannesburg (MJHB), University of Cape Town (UCT), modified University of Cape Town (MUCT), oxic-anoxic (OA) and anaerobic–oxic (A/O). To evaluate the potential of the systems for efficient mitigation of the eutrophication process, the following treated wastewater parameters were included in the simulation: total nitrogen (TN), Kjeldahl nitrogen (N_Kj_), organic nitrogen (ON), ammonium nitrogen (N–NH_4_), nitrate nitrogen (N–NO_3_), nitrite nitrogen (N–NO_2_), total phosphorus (TP), organic phosphorus (OP) and total soluble reactive phosphorus (P_i_) (as orthophosphates P–PO_4_).

In the simulation of the operation of the 10 different systems, the baseline set of input values was systematically modified at constant influent quality parameters, flow intensity and total volume of the bioreactor. The volume of internal sections of the bioreactor with different oxygen conditions, recirculation setup and recirculation rate were modified according to other studies (Henze et al. 2008; Liwarska-Bizukojć 2014; Van Loosdrecht et al. 2015).

### Additional phosphorus post-precipitation

In order to improve the phosphorus removal efficiency, a post-precipitation simulation (introduction of precipitant before the secondary settling tank) was conducted with the use of PIX 111 in the form of liquid iron chloride (III)—FeCl_3_, with 12% of clear iron and density of 1500 kg m^−3^, which is a solution widely used to enhance the elimination of phosphorus compounds (Silvino and Barbosa 2015). The PIX introduction point was set so as to minimize dosage in order to obtain less sewage sludge as well as to allow for the possibility of using secondary settling tank capacity for wastewater final clarification (Aboulhassan et al. 2006; Nowacka et al. 2014; Smol 2018).

For each system analyzed, the dose of precipitant was calculated stoichiometrically based on the phosphorus load in the biologically treated wastewater. This was in accordance with recommendations of Bashar et al. (2018) and required effluent TP content of 1 mg L^−1^. The precipitant dose was calculated according to Formula (3) with the ratio: 1.8 gFe:1gP (Jiang et al. 2004; Kroiss et al. 2011).

3 $$K = Q \cdot \frac{{TP_{\text{EBNR}} - TP_{\text{req}} }}{1000} \cdot S \cdot n,$$

where *K* is the PIX dose (L day^−1^), *Q* is the raw wastewater flow intensity (m^3^ day^−1^), *TP*_req_ is the total permissible phosphorus content in treated wastewater (mg L^−1^), *TP*_ENBR_ is the total phosphorus content after EBNR (mg L^−1^), *S* is the stoichiometric coefficient for FeCl_3_, and *n* is the excess precipitant coefficient.

The simulation of nutrient removal processes in the 10 biological treatment and phosphorus precipitation systems was carried out for 4 different PIX doses calculated as the consistent increase of the calculated dose (a total of 40 variants).

Phosphorus content in biologically treated wastewater without chemical precipitation as well as precipitant doses (k1–k4) used in the simulation for each of the analyzed systems are presented in Table 2.

**Table 2:** TP content without chemical precipitation and calculated PIX doses

Technology	TP after EBNR (mg L^−1^)	PIX calculated dose (k1), (L day^−1^)	2* PIX calculated dose (k2), (L day^−1^)	3* PIX calculated dose (k3), (L day^−1^)	4* PIX calculated dose (k4), (L day^−1^)
CAS	2.76	308	616	924	1232
Anoxic–oxic	2.77	310	619	929	1239
Bardenpho (3-step)	2.24	217	434	651	868
Bardenpho (5-step)	2.48	257	514	771	1029
JHB	1.39	68	136	204	273
MJHB	1.39	68	136	204	273
UCT	1.93	162	325	488	651
MUCT	1.85	148	297	446	595
Anaerobic/oxic	1.79	138	276	414	553
Oxic–anoxic	2.83	320	640	960	1281

The asterisk was supposed to suggest the multiplication sign, as the PIX doses were: double (column 4), triple (column 5), quadruple (column 6)

After the precipitant doses have been established a dynamic simulation of the bioavailable nutrient content was carried out.

## Results and discussion

### Phosphorus forms content

**Fig. 3:**
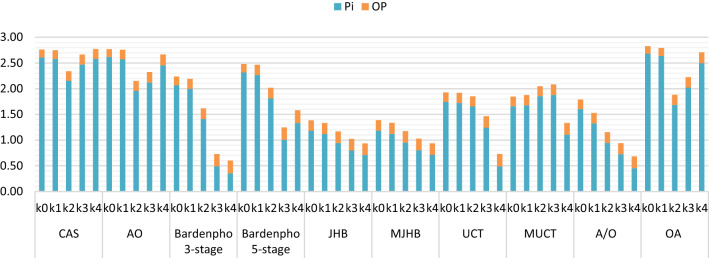
Effluent phosphorus content in the analyzed treatment systems (mg L^−1^), where P_i_—total soluble reactive phosphorus, OP—organic phosphorus, k0—the simulation variant without chemical phosphorus precipitation, k1–k4 are the PIX doses according to Table 2

The aim of the research presented in this paper is to develop a key tool for selection of appropriate wastewater treatment technologies. To achieve this aim, simulation tests were carried out to identify the systems providing the lowest level of EPTW for both nitrogen and phosphorus limited eutrophication in receiving water bodies. Simulation results of phosphorus removal in the examined technologies are shown in Fig. 3.

Based on the results of the ASM-based simulation, the lowest P_i_ content in treated wastewater was obtained in a chemically supported 3-stage Bardenpho system (0.35 mg L^−1^). A slightly higher P_i_ content was obtained in the following technologies: A/O (0.46 mg L^−1^), UCT (0.49 mg L^−1^) and JHB and MJHB (0.71 mg L^−1^) systems. The rest of the analyzed technologies were characterized by an unsatisfactory P_i_ content exceeding the required 1.0 mg L^−1^. Results for these technologies were as follows: 5-stage Bardenpho (1.01 mg L^−1^), MUCT (1.11 mg L^−1^), OA (1.68 mg L^−1^), AO (1.96 mg L^−1^) and CAS (2,16 mg L^−1^).

The OP content was less variable (0.20 ± 0.05 mg L^−1^). The lowest value (0.15 mg L^−1^) was obtained with the CAS, AO and OA systems without chemical precipitation. The highest (0.25 mg L^−1^) was achieved in the systems with the highest efficiency of P_i_ removal: 3 and 5-stage Bardenpho technology.

Excessive phosphorus content is commonly thought to be the main cause of eutrophication in freshwaters and primary production in the world’s oceans (Schindler 2006). Within the phosphorus removal simulation, a predominance of P_i_ over OP was observed in all of the investigated systems. The P_i_/OP ratio gradually decreased following increased PIX doses as a high share of P_i_ in TP content has been proved to have a stimulating effect on phytoplankton and planktonic bacteria growth (Vinçon-Leite and Casenave 2019). Moreover, many studies suggest that the P_i_/OP ratio may vary depending on the different wastewater treatment technologies and the precipitation chemicals actually used (e.g., biological processes, membranes and ferric or aluminum salts) (Brett and Li 2015).

### Nitrogen forms content

**Fig. 4:**
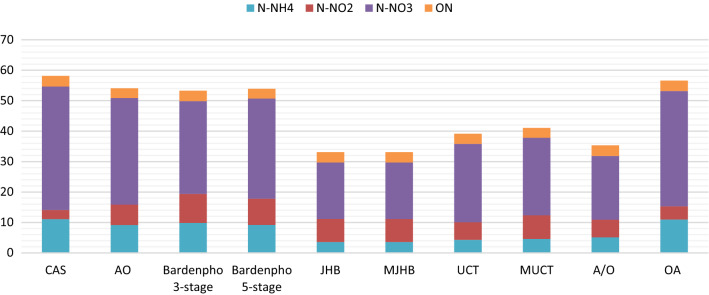
Effluent nitrogen content in the analyzed treatment systems (mg L^−1^)

Besides phosphorus, nitrogen compounds can also cause limited eutrophication, especially in shallow lakes and marine waters (Phillips et al. 2008). Therefore, analogously to the above, an ASM simulation of nitrogen compound removal efficiency was carried out. Results are shown in Fig. 4.

The lowest simulated content of total inorganic nitrogen (TIN) (measured as the sum of N–NH_4_, N–NO_2_ and N–NO_3_) was identified in the JHB and MJHB systems (both giving 29.71 mg L^−1^), while wastewater treated by A/O, UCT and MUCT was characterized by TIN values of: 31.85 mg L^−1^, 35.83 mg L^−1^, 37.81 mg L^−1^, respectively.

The highest TIN content in treated wastewater was identified in the CAS system (54.66 mg L^−1^). However in the OA, AO, 3-stage and 5-stage Bardenho systems TIN content reached the following values of 53.18 mg L^−1^, 50.89 mg L^−1^, 50.73 mg L^−1^, 49.85 mg L^−1^, respectively. These results also exceeded the required standards.

Values for ON content varied from 3.17 mg L^−1^ in the 5-stage Bardnepho system up to 3.49 mg L^−1^ in CAS technology with the rest of the analyzed systems giving ON concentrations of AO—3.19 mg L^−1^, UCT and MUCT—3.24 mg L^−1^, JHB and MJHB—3.39 mg L^−1^, 3-stage Bardenpho and OA—3.41 mg L^−1^, A/O—3.48 mg L^−1^.

A substantial difference between the simulated and measured nitrogen content was observed within the study. The most likely explanation of this can be the fact that at the Krakow-Kujawy WWTP a facultative chamber is used within the bioreactor. This facultative chamber can operate as an oxic or anoxic chamber according to actual process needs and raw wastewater composition. The operating time of the facultative chamber in oxic or anoxic conditions was not available and therefore it was not included in the simulation input data.

It should be noted that in the case of nitrogen, the TIN/ON ratio may also have significant importance (Vaiopoulou and Aivasidis 2008; Zhou et al. 2018). A high TIN/ON ratio in treated wastewater can directly trigger eutrophication acceleration (Li and Brett 2012) as well as effluents characterized by a low TIN/ON ratio which can also cause acceleration of eutrophication in sensitive estuaries and inland waters (Schindler 2006; Huang et al. 2017; Biswas et al. 2018).

### Evaluation of bioavailable nutrient share in wastewater

The next research stage was to realize the main objective of EPTW assessment based on its definition as formulated by the authors. In this stage, the technologies studied were assessed in terms of bioavailable nutrient removal efficiency and EPTW level. To evaluate the impact of wastewater discharged from analyzed wastewater treatment systems on eutrophication development, the share of bioavailable (inorganic) forms in TN and TP content was evaluated. The values of BAN% and BAP% calculated according to Formulas 1 and 2 are presented in Table 3.

**Table 3:** The values of BAN% and BAP% obtained in the simulation, where k0—the simulation variant without chemical phosphorus precipitation, k1–k4 are the PIX doses according to Table 2

Technology	PIX dose	BAN%	BAP%	Technology	PIX dose	BAN%	BAP%
CAS	k0	94	95	MJHB	k0	90	85
	k1	–	94		k1	–	84
	k2	–	92		k2	–	81
	k3	–	93		k3	–	78
	k4	–	93		k4	–	76
AO (anoxic–oxic)	k0	94	95	UCT	k0	91	90
	k1	–	93		k1	–	90
	k2	–	91		k2	–	89
	k3	–	91		k3	–	85
	k4	–	92		k4	–	67
3-Stage Bardenpho	k0	94	92	MUCT	k0	92	90
	k1	–	91		k1	–	89
	k2	–	87		k2	–	91
	k3	–	67		k3	–	90
	k4	–	58		k4	–	83
5-Stage Bardenpho	k0	94	94	A/O (anaerobic/oxic)	k0	90	90
	k1	–	92		k1	–	87
	k2	–	90		k2	–	82
	k3	–	81		k3	–	77
	k4	–	84		k4	–	67
JHB	k0	90	85	OA (oxic–anoxic)	k0	94	95
	k1	–	84		k1	–	94
	k2	–	81		k2	–	89
	k3	–	78		k3	–	91
	k4	–	76		k4	–	92

Reviewing the simulation results summary, a recommendation that comes to mind is that, in cases where the eutrophication process is limited by phosphorus compounds in the wastewater receiver, the most appropriate technologies providing the lowest EPTW could be chemically supported 3-stage Bardenpho (BAP% = 58%), A/O and UCT (BAP% = 67%), JHB and MJHB (BAP% = 76%). When treated wastewater is discharged into a recipient where eutrophication is limited by nitrogen compounds, the recommended wastewater treatment technologies are the following: JHB, MJHB and A/O (BAN% = 90%), UCT and MUCT (BAN% = 91% and 92%, respectively).

### Implementation concept of a dual recipient-oriented approach

In order to identify operation methods to solve the complex problems of eutrophication process management, the following key aspects should be distinguished:

1. Advanced wastewater treatment technologies can theoretically provide exceptionally high efficiencies for pollution reduction; however, treatment costs increase exponentially and the community is often unable to bear such financial burdens (Jiang et al. 2004; Nedelciu et al. 2019).
2. With the increasing complexity of technological systems and the intensification of the applied processes, environmental pressure caused mostly by increased energy consumption and greenhouse gas emissions increases significantly (Henriques and Catarino 2017; Castellet-Viciano et al. 2018).
3. Approaches to the development of treated wastewater quality standards should take into account the response of the aquatic ecosystem to the introduced pollutant loads including the receiver properties and their sensitivity to eutrophication (Cramer et al. 2018; Poikane et al. 2019a; Rogowska et al. 2019).
4. Selection of wastewater treatment technologies should be based on EPTW analysis with consideration of the key eutrophication limiting factors in separate receivers.
5. ASM simulation can be an efficient tool (Guerrero et al. 2013) for supporting decision-making processes regarding selection of an appropriate wastewater treatment technology with a low EPTW.

**Fig. 5:**
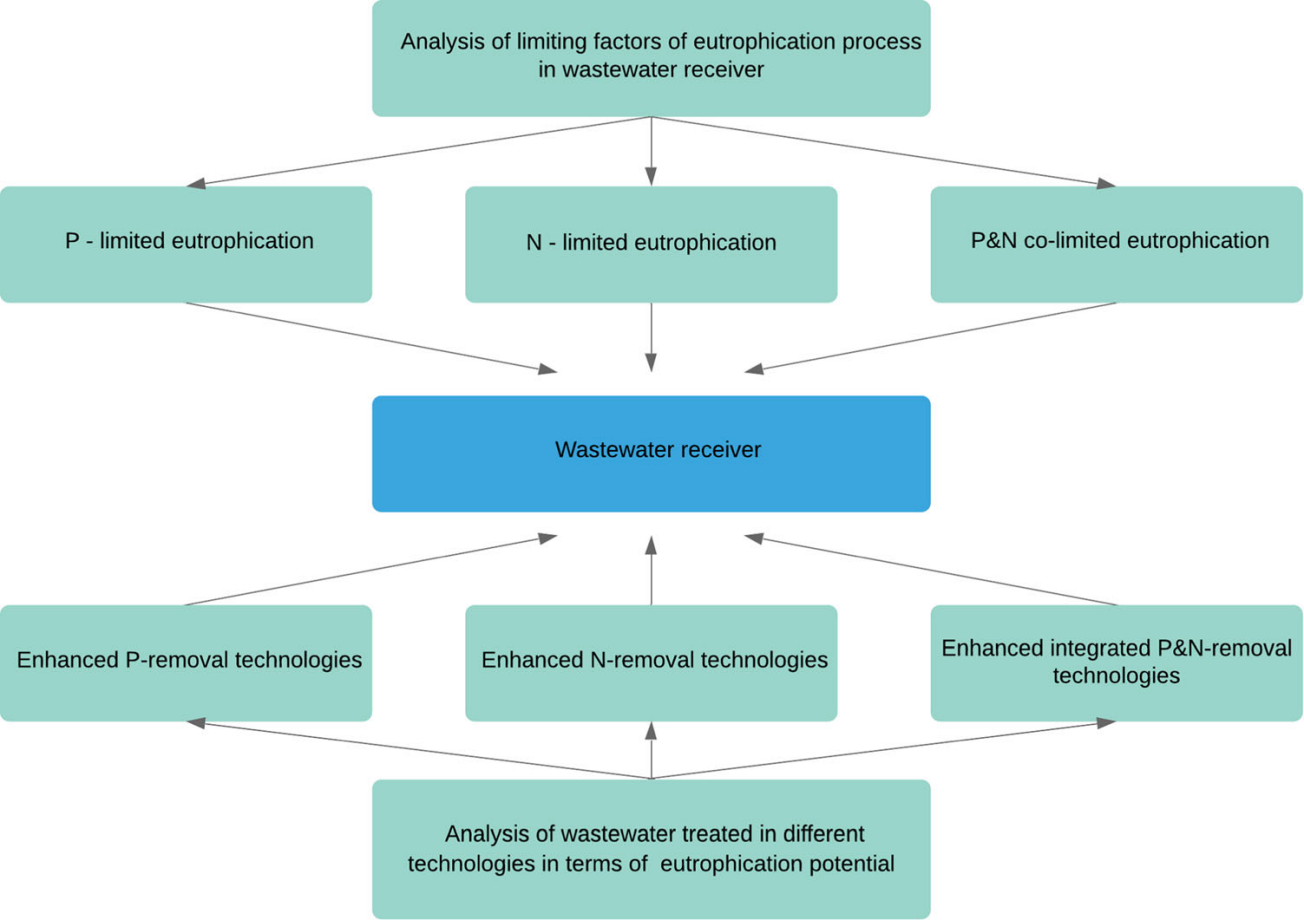
Implementation concept of the proposed approach

In order to improve surface water state by ensuring an optimal quality of treated wastewater, a dual recipient-oriented approach has been developed. The conceptual scheme of the proposed approach is shown in Fig. 5.

A special emphasis was put on harmonizing treated wastewater quality standards and receiving ecosystem response for instance by analyzing the eutrophication limitation factors in particular recipients and by the selection of appropriate nutrient removal technologies based on their EPTW. Through consideration of the key limiting factor when selecting treatment technologies with adequate nutrient removal efficiency and low EPTW, it is possible to make ecologically sound and economically justified decisions.

The approach proposed corresponds with the results of other research, e.g., Poikane et al. (2019a, b), suggests the implementation of nutrient criteria based on actual biological responses to nutrients. Water ecosystem response to nutrient loading variability is complicated by non-linear feedback mechanisms, so we should review and clarify our understanding of specific systems (Viktorsson et al. 2013). It should be also taken into account that increased phytoplankton production associated with nutrient enrichment leads to bottom-water oxygen depletion, which changes the fundamental nature of sediment biogeochemistry and nutrient cycling, increasing the rate and efficiency of their recycling from sediments enhancing eutrophication (Håkansson and Bryhn 2008). A central question to the application of scientific understanding to eutrophication mitigation is the relative importance of nitrogen and phosphorus as nutrients limiting the primary production (Kemp et al. 2005).

Several planned nutrient stream mitigation measures to limit eutrophication and restore the nutrient balance in the North Sea coastal zone were tested by Thieu et al. (2010). The results of this model-based analysis indicated that a 47–72% reduction of point-source phosphorus discharges could be reached if intensive phosphorus treatment was applied in the largest WWTPs in the analyzed catchment area, while a 14–23% nitrogen content reduction is possible through an integrated approach consisting of advanced wastewater treatment and management measures aimed at regulating agricultural practices.

Moreover, the need to reconsider the current eutrophication mitigation approach in its legal and technical aspects is highlighted by the integrated eutrophication status assessment developed by the Helsinki Commission (HELCOM) for 2011–2016, which confirmed that up to 97% of the Baltic Sea waters are still in the treat of eutrophication (HELCOM 2016). Despite the reduction of total nutrient loads was achieved, only 17 from 247 coastal and open water bodies status was assessed as “good” (HELCOM 2015). Furthermore, according to HELCOM analysis, none of the Baltic States achieved the nutrient maximum allowable inputs (MAIs) in all waters particularly affected by eutrophication (Baltic Proper, Gulf of Finland and Gulf of Riga) (Svendsen et al. 2015).

Understanding of eutrophication mechanisms as a key element to stop the development of this process can be found in many publications. Results of the study by Wang and Wang (2009) carried on over 40 Yangtze lakes proved that phosphorus is the key factor determining phytoplankton growth regardless of nitrogen concentrations.

The eutrophication studies in the Potomac River estuary have shown that the frequency of toxic cyanobacteria summer blooms declined sharply in the early 1970s when phosphorus removal from wastewater was introduced, although other harmful algal blooms (HABs) in the Chesapeake Bay appeared to be stimulated by the discharge of dissolved organic nitrogen (DON). In addition, the role of nitrogen and phosphorus as limiting factors in the Chesapeake Bay exhibits well-defined seasonal and regional variations (Kemp et al. 2005).

Restrictive legal measures were applied in the Seto Inland Sea in Japan which was affected by serious eutrophication in the 1960s and 1970s caused by intensive municipal and industrial wastewater discharge. The introduction of the “Law Concerning Special Measures for Conservation of the Environment of the Seto Inland Sea” resulted in a decrease of nutrients loads discharged to the Seto Inland Sea which enabled to lower the surface and middle water layer concentration of P–PO_4_ from 0.7 µM in 1979 to 0.2 µM in 1984, N–NO_3_ from 5.9 µM in 1979 to 1.0 µM in 1983 and N–NH_4_ from 4.9 µM in 1974 to 0.8 µM in 1981 preventing the HABs occurrence (Imai et al. 2006).

The results of other research undermine traditional opinion regarding the necessity of simultaneous removal of both nutrients without consideration of limiting factors (Lee et al. 1978; Petzoldt and Uhlmann 2006).

The research presented, as well as the present study, shows that without a deep analysis of eutrophication limiting factors and wastewater treatment technology performance it is difficult to provide efficient and ecologically acceptable protection against eutrophication.

## Conclusions

In the light of the results presented in this paper, it can be assumed that current approaches to the selection of nutrient removal technologies designed for eutrophication mitigation caused by wastewater discharge do not take into account the nutrient compound bioavailability and, thus, need to be reconsidered in their legal and technical aspects. The simulation results for nutrient content in treated wastewater obtained in various activated sludge technologies are useful for evaluating the level of WWTP impact on eutrophication development based on the simulated EPTW. The results suggest that the primary application of chemically supported 3-stage Bardenpho, A/O, UCT, JHB or MJHB systems when eutrophication in effluent receiving water body is limited by phosphorus. Furthermore, when nitrogen is the eutrophication limiting factor we suggest using primarily JHB, MJHB, A/O, UCT or MUCT systems. However, the proposed approach to eutrophication mitigation could change the current situation only if the common perception focused on the total loads of nutrients will be modified and the bioavailability context including eutrophication limiting factors gains much more awareness. Otherwise, even the most advanced, energy-intensive wastewater treatment technologies will not be able to mitigate eutrophication.
